# A network and mediation analysis on the associations between family environment and suicidal ideation in adolescents in a psychiatric clinical setting

**DOI:** 10.3389/fpsyt.2025.1541993

**Published:** 2025-03-07

**Authors:** Xing-Yan Liu, Shu-Hui Xu, Wen-Jing Yan, Li-Li Zhu, Cheng-Han Li

**Affiliations:** ^1^ Department of Psychiatry, Wenzhou Seventh People’s Hospital, Wenzhou, China; ^2^ Career Education Research Center, Wenzhou University, Wenzhou, China; ^3^ Emergency Department, Third Affiliated Hospital of Wenzhou Medical University, Wenzhou, China

**Keywords:** suicidal ideation, family environment, adolescents, network analysis, mediation analysis

## Abstract

**Background:**

Family environmental factors are known to contribute to adolescent suicidal ideation (SI), but how these factors interact and relate to SI needs further investigation.

**Aims:**

To examine how family factors interact with each other and are associated with adolescent SI in a psychiatric clinical setting, using network analysis with regularization methods.

**Method:**

Utilizing a quantitative research design, this study analyzed data from 293 adolescents aged 12 to 18 years seeking care in a psychiatric hospital. Data collection involved standardized interviews and self-report measures to assess SI, anxiety, depression, and various family environmental factors. Network analysis with regularization methods, including LASSO regression, was employed to elucidate the relationships among these variables.

**Results:**

Over 40% of adolescents reported SI, with positive relationship quality(RQ) significantly reducing SI. Network analysis indicated that family economic status did not directly relate to SI but through RQ. Additionally, anxiety was found to mediate the relationship between RQ and SI significantly, with a mediation effect of 53.34%. Parental marital status directly related to SI, whereas parental education level, particularly mothers’, was not directly associated with SI or other mental health outcomes.

**Conclusions:**

This study reveals the complex interplay between family environmental factors and psychiatric symptoms in adolescents, highlighting family relationship quality as a critical risk mechanism. These findings underscore the importance of family-centered interventions and public mental health policies to reduce suicidal ideation in adolescents.

## 1 Introduction

Suicide is a major public health concern and the second leading cause of death among young people worldwide (1). Suicidal ideation (SI) is a common precursor to suicide attempts. The lifetime prevalence of SI begins to rise in early adolescence and increases rapidly in late adolescence (2), ranging from about 10% to 40% for suicidal ideation, depending on the specific population and setting (3). Based on the Global School-based Student Health Survey (GSHS) of adolescents aged 12-17 years in 82 districts in six World Health Organization (WHO) regions between 2003 and 2015, the prevalence was found to be 14% (4).

Higher rates have been reported in adolescents in psychiatric settings. For example, a study by Neupane and colleagues found that 38.2% of adolescents admitted to psychiatric hospitals reported a history of suicide attempt (5). Some studies have reported SI in patients with depression, reaching approximately 50% (6). The higher rate of SI reported by adolescents admitted to psychiatric hospitals may be due to their mental health problems, such as mood disorders, which have emerged as one of the strongest predictors of suicidal ideation and behavior. Two lines of evidence suggest a strong association between psychiatric disorders and suicide risk (7). First, psychiatric autopsy studies have found that 67% to 95% of adolescents who die by suicide have a diagnosable psychiatric disorder at the time of the attempt (8). Second, these findings have been supported by the results of a number of controlled studies that include non-suicidal individuals. These studies have consistently shown higher rates of psychiatric disorders among youth with suicidal ideation and attempts (9).

Therefore, it is important to better understand and predict the factors that are associated with SI in adolescents, especially in the clinical setting of psychiatry, where much higher rates are found. Many studies have traced mental health problems to relatively distant risk factors and have found an important role for family environmental factors (9), which may be independently associated with SI. In the present study, we sought to examine how these factors interact and are associated with SI in adolescents in a psychiatric clinical setting.

### 1.1 Relationship quality

Previous research suggests that poor relationship quality (RQ) is a particularly important family environment factor that may influence the development of SI during adolescence, and that high positive RQ may buffer those at risk for SI. Parent-child relationships characterized by high levels of positive qualities have been shown to be protective for adolescents at high risk for suicide (10). Positive parenting (e.g., warmth, responsiveness) and negative parenting (e.g., control, hostility) are related to Chinese adolescents’ suicidal ideation (11). Another study showed that parent-child RQ predicts adolescent SI and suicide attempts, including four studies of representative samples from the same country (12). Specifically, family functioning, parental attachment, and family cohesion have been found to be associated with suicidal ideation (11).

### 1.2 Parental marital status

Research suggests that parental marital status may be an important factor in adolescent suicidal ideation. A meta-analysis of 54 studies found a strong association between parental divorce and suicidal ideation (unadjusted OR = 1.81, 95% CI = 1.49 to 2.14, p <.001) (13). Studies have consistently shown that adolescents from divorced or single-parent families are more likely to experience suicidal ideation than those with married parents (14). Studies have also found that adolescents who have experienced family breakups, such as divorce or separation, are more likely to report SI than those who have not experienced such breakups (15). In addition, traditional Chinese values reject divorce as a shameful event. As a result, parents may prefer to struggle to stay together rather than divorce. This preference may lead to poor parental relationships. Poorer parental relationships and higher divorce rates bring misfortune and psychological distress to students, which may lead to suicidal thoughts.

### 1.3 Parental economic status

Research has consistently shown a strong relationship between family economic level and suicidal ideation (16). Many studies over the past decade have reached the same conclusion. Cuesta and colleagues (17) found that teenagers of lower family income report high percentages of suicidal thinking or suicide attempts. A recent study found that parental occupational status was significantly associated with suicidal ideation among Korean adolescents (18). The study compared the relationship between parental income and suicidal ideation among 3201 adolescents aged 12-18 years. It found that adolescents’ SI was associated with parents’ employment status, work status, work schedule patterns, and working hours per week. lower parental income was associated with higher levels of suicidal ideation, especially father’s occupational status.

### 1.4 Parental education

Parental education level can also be a risk factor for adolescent suicidal ideation. In a study, those with low parental education levels were more likely to report suicidal ideation than their peers with higher parental education levels (19). This finding was further supported by a study which found that adolescents with lower parental education levels were more likely to consider suicide than their peers with higher parental education levels (20). A meta-analysis included a total of 59 articles shows that lower parental education was associated with youth suicidal attempts but geographical region and country income level moderated the associations (21). Lower parental education was associated with an increased risk of youth suicidal attempts in Northern America (OR = 1.26, 95% CI = 1.10–1.45), but with a decreased risk in Eastern and South-Eastern Asia (OR = 0.72, 95% CI = 0.54–0.96). The relationship between parental education and SI has been inconsistent in previous studies and may depend on different circumstances.

### 1.5 Investigating the connections between factors

There are many variables that influence suicidal ideation, and it is useful to identify important variables and compare their effects. Because these variables are often inextricably linked, it is necessary to control for variables to show the substantial effects of a variable’s predictor variables on the outcome variables. Many studies have attempted to do this by controlling covariates or using partial correlations. However, these traditional methods do not provide a complete picture. Network analysis, which has been developed in recent years, is a useful tool for analyzing relationships between variables, including psychological characteristics and symptoms (22). For example, one study (23) applied network analysis to assess the interconnections between psychological symptoms in suicidal individuals; another network (24) analysis study revealed that loneliness was both a central symptom in depression networks and the strongest predictor of suicidal ideation. These studies underscore the value of network analysis in identifying key variables and pathways in suicide research, providing a strong foundation for its application in the current study. In this study, demographic variables and symptoms were included alongside family environment factors to explore their complex interactions. The network provides a panoramic visualization of variable relationships and allows us to look from the whole to the local (22). Therefore, network analysis is a very good way to present our cross-sectional data. Furthermore, network analysis often uses LASSO regression (25), which can effectively prevent overfitting, which is very common in psychological and psychiatric research. Based on network analysis, we will conduct a path analysis to zoom in, to find the direct and indirect effects.

In summary, family environmental factors are thought to contribute to adolescent suicidal ideation (SI). However, how these factors interact dynamically and are associated with SI remains unclear, particularly in psychiatric clinical settings where multiple risk factors coexist. This study employs a network analysis approach to explore the relationships among family environmental factors, anxiety, depression, and SI. Specifically, we aim to investigate (1) how family relationship quality (RQ), parental marital status, family economic status, and other family-related factors interact with adolescent anxiety, depression, and SI, and (2) the mediating role of anxiety and depression in the relationship between family factors and SI.

## 2 Method

### 2.1 Participants

The sample consisted of 293 adolescents aged 12 to 18 years who sought clinical care at a psychiatric hospital between February and July 2022. Participants were recruited from a larger pool of adolescents attending the hospital during the study period, using a consecutive sampling strategy. Inclusion criteria were being between the ages of 12 and 18 and willingness to participate in the study. Exclusion criteria included severe cognitive impairment (assessed in the clinician’s interview) and an inability to complete the questionnaires. Each participant completed a hospital registration process followed by an individual mental health assessment by a hospital psychiatrist. Prior to the mental health assessment, the patient and parent/guardian were briefed about the study’s objectives, risks, and benefits. Written informed consent was obtained from the parent/guardian, and assent was obtained from the adolescent participant. Both the informed consent and assent were required as per the local hospital ethics regulations. In addition to the usual diagnostic process, patients were assessed by the clinician using the Self-Injurious Thoughts and Behaviors Interview (SITBI) and asked to complete the Hospital Anxiety and Depression Scale (HADS).

### 2.2 Measures

#### 2.2.1 Demographical information

For each patient we collected basic demographic information, including sex, age, household registration (provincial capital city, non-capital city, township, rural area), whether or not they were an only child, parents’ education (illiterate, primary, junior high, high school, college, master’s degree and above), parents’ marital status (intact, single parent, reconstituted, orphaned), family relationships (close, cordial, distant, strained, conflicted), family economic status (rich, better off, average, poor), and family psychiatric history. For simplifying the variable for network analysis, family relationships were dichotomized into ‘positive’ (combining close and cordial relationships) and ‘negative’ (combining distant, strained, and conflicted relationships); ‘rich’ and ‘better off’ were grouped into the ‘wealthy’ category, while ‘average’ and ‘poor’ were grouped into the ‘average/poor’ category.

#### 2.2.2 Self-Injurious Thoughts and Behaviors Interview (SITBI)

The SITBI is a structured interview that assesses the presence, frequency, and characteristics of various self- injurious thoughts and behaviors, including SI, suicide planning, suicidal gestures, suicide attempts, and non-suicidal self-injury (26).

#### 2.2.3 Hospital Anxiety and Depression Scale

The Hospital Anxiety and Depression Scale (HADS) is a 14-item self-report measure that assesses anxiety and depressive symptoms, focusing on nonphysical symptoms. It comprises two subscales: HADS-A for anxiety and HADS-D for depression. The scale is known for its simplicity and ease of administration. Its internal consistency is robust (27), with a mean Cronbach’s alpha of.83 for the HADS-A and.82 for the HADS-D. In our dataset, the Cronbach’s alpha for the Anxiety subscale was 0.878, and the Depression subscale was 0.857.

### 2.3 Network analysis

Network analysis allows the visualization and quantification of relationships between variables in a complex system. In the field of psychology, network models represent symptoms or characteristics as nodes, and their interactions as edges connecting nodes (28). One common approach is to use Markov Random Fields (MRFs) to estimate conditional independence relationships between variables that can then be represented in an undirected network graph (29).

In this study, we conducted network analysis using the *mgm* package (v1.2) in R, which can estimate MRFs for mixed variable types, including both continuous and categorical variables. The *mgm* package determines conditional independence using regression analysis with regularization methods. Specifically, it employs lasso (L1) and ridge (L2) regularization provided by the *glmnet* package (v4.1). Regularization helps reduce overfitting that often occurs with highly collinear predictors in psychological data (25). The weight values in our network represent standardized regression coefficients obtained through the mgm package’s mixed graphical model estimation. The lambda parameter controls the overall strength of regularization. We specified lambdaGam = 0.25, which was determined through preliminary sensitivity analyses. This approach is consistent with recommendations in the literature to empirically select lambda values that optimize network stability and interpretability (25). In addition, an undirected network graph can be plotted to visualize the relationships (*qgraph*, v1.9).

## 3 Results

### 3.1 Descriptive statistics

Descriptive statistics for the measurements on 293 adolescents in psychiatric settings are found in **Table 1**. Parents’ marital status (intact vs. irregular), economic status (wealthy vs. average/poor), RQ (positive vs negative), location (urban vs. rural) were coded as binary variables. The decision to employ binary coding was driven by the nature of our research questions and the balance of the sample distributions.

**Table 1:** Descriptive statistics for the measurements on 293 adolescents in psychiatric settings.

		Suicidal ideation		Anxiety		Depression	
		Negative (*N*)	Positive (*N*)	*M*	*SD*	*M*	*SD*
Sex	Female	78	77	8.46	4.59	7.28	4.63
	Male	85	53	6.57	4.60	6.19	4.27
Marital status	irregular	13	33	9.30	5.12	9.22	4.26
	intact	150	97	7.25	4.54	6.31	4.39
Economic Status	average/poor	110	101	8.12	4.78	7.22	4.48
	Wealthy	53	29	6.17	4.13	5.60	4.32
Only Child	Siblings	130	98	7.50	4.62	6.55	4.40
	Single	33	32	7.83	4.92	7.51	4.76
Relationship Quality	Negative	19	51	10.69	4.25	9.71	4.60
	Positive	144	79	6.60	4.39	5.84	4.04
Father Education	Illiterate	1	0	6.00		6.00	
	Primary	24	25	8.14	5.36	7.71	5.24
	Junior high	82	66	7.45	4.52	6.78	4.37
	High school	45	23	7.00	4.75	5.44	3.93
	College	11	16	8.74	4.09	8.33	4.35
Mother Education	Illiterate	0	2	11.50	2.12	11.50	.71
	Primary	29	22	6.76	3.86	6.14	3.75
	Junior high	74	55	7.62	4.79	6.98	4.71
	High school	41	38	8.13	4.79	6.80	4.56
	College	18	13	7.23	5.20	6.42	4.60
	Master’s or above	1	0	2.00		10.00	
Location	Rural	78	57	7.08	4.77	6.27	4.77
	Urban	85	73	7.99	4.59	7.18	4.21

### 3.2 Network analysis

**Figure 1:**
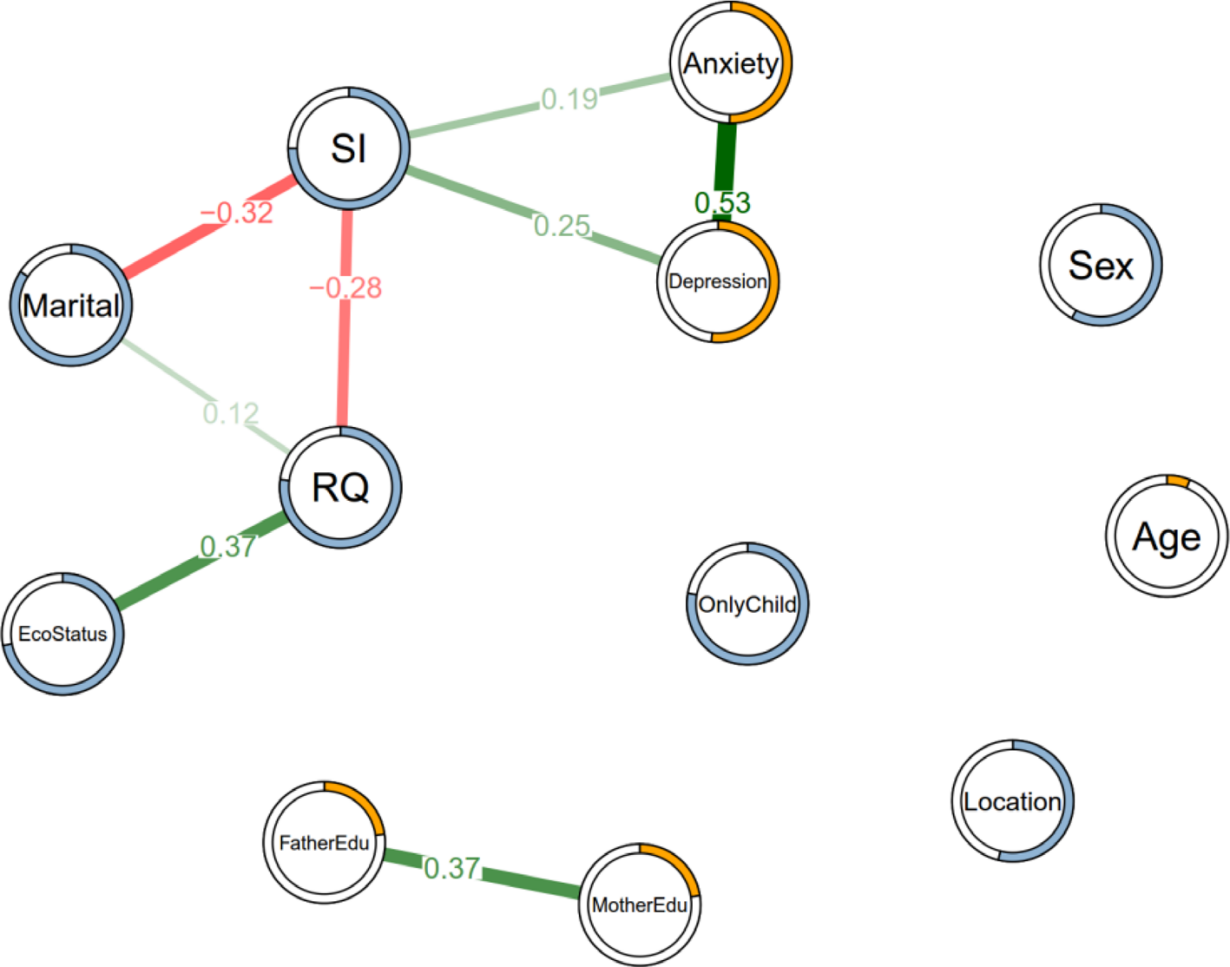
Estimated network structure of 293 adolescents based on the *mgm* package in R. Green/red edges indicate positive/negative weights (coefficients) between nodes. With LASSO regression, some coefficients between the variables were squeezed to zero. Rings on nodes indicate R^2^ (continuous variable, orange) or categorical accuracy (categorical variable, light blue).

In the network, 58 of the 66 network edges are set to zero by the regularization. **Figure 1** shows the structure of the estimation. The values on the edges show the weights between the nodes; thicker edges imply stronger relationships. The structure shows that family economic status is not directly related to suicidal ideation (SI), but affects it through RQ (weight = 0.37). RQ and parental marital status were directly related to SI with weights of -0.28 and -0.32, respectively, indicating that positive RQ and intact parental marital status reduce SI in these adolescents. SI was highly associated with mood disorders such as anxiety (weight = 0.19) and depression (weight = 0.25). Parental education level was highly correlated, but not with other variables. After regularization, sex, age, being an only child, and location were not associated with SI, anxiety, and depression. Network centrality indices were not presented in this study because they were not meaningful for these rather heterogeneous variables.

We initially considered three standard centrality measures: strength, betweenness, and closeness. We don’t provide the centrality indices here because (1) the variables in the network are of mixed types, which makes the computation and interpretation of centrality measures less straightforward and potentially misleading and (2) The nodes in the network are not on the same level (demographical vs. psychological), further complicating the meaningfulness of centrality indices in this context. Similarly, the stability analysis of the centrality was omitted.

### 3.3 Mediation effects analysis

Based on the previous network analyses, we conducted a mediation effects analysis of the local network to further validate the relationship between the variables.

**Figure 2:**
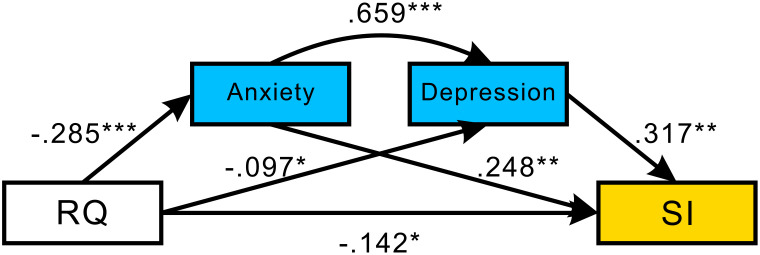
The serial mediation analysis from RQ to SI mediated by anxiety and depression. In addition, we use RQ as a mediator, i.e., economic status → RQ → SI, to examine the relationship between economic status and SI. We found that the direct effect was insignificant, *β*=-0.134, *p*=0.403, while the indirect effect was significant, *β*=-0.188, *p*<0.001. *p < 0.05 (statistically significant at the 5% level), **p < 0.01 (statistically significant at the 1% level), ***p < 0.001 (statistically significant at the 0.1% level).

First, to test whether anxiety and depression mediated the association between RQ and SI, we conducted a path analysis using weighted least squares (WLSMV) estimation implemented in Mplus version 8.3 (see **Figure 2**). Background variables were controlled for sex, age, whether only child, economic status, maternal education level, paternal education level, and location. The results in **Table 2** show a significant overall effect of RQ on SI (overall effect = -0.30, 95% CI [-0.439, -0.167]). The direct effect of RQ on SI is -0.14, 95% CI [-0.276, -0.009]. A significant mediating effect of anxiety was found (indirect effect = -0.07, 95% CI [-0.129, -0.013]), with a negative association between RQ and anxiety (*β* = -0.285, *p* < 0.001) and a positive association between anxiety and SI (*β* = 0.248, *p* = 0.008). However, the indirect effect of depression was not significant (95% CI [-0.065, 0.004], including zero). Overall, the mediating effect of anxiety and depression in the pathway from RQ to SI was 53.34% (0.16/0.30 [standard estimate of the total mediation effect/standard estimate of the total effect]).

**Table 2:** Summary of direct and indirect effects of the mediation model.

Paths	Effects	S.E.	*t*	*p*	Bootstrapping 95% CI
Indirect effect					
RQ→Anxiety→SI	-0.07	0.03	-2.39	0.017	[-0.129, -0.013]
RQ→Depression→SI	-0.03	0.02	-1.75	0.080	[-0.065, 0.004]
RQ→Anxiety→Depression→SI	-0.06	0.02	-2.52	0.012	[-0.106, -0.013]
Direct effect					
RQ→SI	-0.14	0.07	-2.09	0.037	[-0.276, -0.009]
Total effect	-0.30	0.07	-4.38	<0.001	[-0.439, -0.167]

RQ, Relationship quality; SI, Suicidal ideation; CI, Confidence interval; *p* < 0.05 was considered significant.

## 4 Discussion

In the present study, suicidal ideation is very common among Chinese adolescents in a psychiatric setting, where over 40% of the participants had suicidal thoughts in the past two weeks, while only about 10% of the general population. We found that positive RQ and intact parental marital status were inversely related to SI, suggesting that supportive family dynamics and stable parental relationships serve as protective factors against the emergence of suicidal thoughts among adolescents. Furthermore, our findings indicated that family economic status was indirectly related SI through its effect on RQ, highlighting the complex ways in which economic factors can be associated to mental health beyond direct financial stress, by affecting the quality of family interactions and support. The mediation effects analysis further elucidated the pathways through which RQ was associated with SI, with anxiety serving as a significant mediator. This suggests that the quality of family relationships not only has a direct effect on SI but also affects adolescents’ mental health, which in turn influences their suicidal ideation (30). This intricate web of relationships underscores the multifaceted nature of SI risk factors and the need for holistic approaches in prevention and intervention strategies that consider the broader family and socio-economic context of adolescents’ lives.

Our findings suggest that RQ may be a key factor in the chain of events associated with suicidal ideation. This is consistent with previous research suggesting that RQ has a significant impact on mental health outcomes and may increase the likelihood of suicidal ideation (31, 32). In this context, it has been suggested that interpersonal relationships form the basis of social support. This is because a lack of social support can lead to increased anxiety, depression and suicidal ideation (33). Thus, our study provides new evidence for the association between RQ and SI, and further highlights the importance of interpersonal relationships as a source of social support. Improving the quality of adolescents’ family relationships could be used as an intervention to reduce the occurrence of suicidal behavior.

However, further research has suggested that RQ may also be a mediation factor in SI (34). Another finding of this study was that anxiety and depression mediated the relationship between RQ and SI. Previous studies also found a strong association between poor mental health outcomes, including anxiety and depression, and SI (35, 36). By identifying anxiety as a mediator, our study not only corroborates these findings but also provides insight into how improvements in RQ could potentially reduce anxiety and, consequently, SI. It can be hypothesized that adolescents with conflicting family relationships receive less social support and are more likely to experience more negative emotions (anxiety and depression) than adolescents with close family relationships, leading to a rapid increase in suicidal ideation in late adolescence.

Furthermore, the results show that economic status was indirectly associated with SI through its relationship with RQ, suggesting that good economic status is associated with better relationships. There is an old Chinese saying that “poor couples have a lot of sorrow”, suggesting that the economic situation is not good, there will be more conflict in the family. However, there are also families that manage to maintain close and warm relationships even without a good financial situation, which is protective for young people’s mental health. This suggests that increasing the frequency and quality of parent-child interactions may be effective in improving adolescents’ mental health and reducing SI, even when the family’s economic situation is less favourable (37).

There is a relationship between RQ and parental marital status. Previous research has shown a strong relationship between marital and family stability and mental health. In this study, the direct effect of parental marital status on SI was analyzed while controlling for demographic and family-related variables, ensuring the results were not confounded by factors such as age, sex, or economic status. Additionally, in Chinese culture, the stigma associated with divorce often prevents families from seeking external help or openly resolving conflicts. This can lead to prolonged familial tensions, indirectly exacerbating adolescents’ vulnerability to SI (38). Addressing such social taboos is critical for reducing emotional distress in adolescents.

Furthermore, this study found no relationship between the mother’s level of education and other variables, including symptoms of suicidal ideation. This result is consistent with some findings (21), who reported that parental education did not influence the risk of suicidal ideation among adolescents in their study. In many Chinese households, mothers often prioritize providing emotional support and maintaining family cohesion over contributing to socioeconomic status, regardless of their educational background. This dynamic may buffer adolescents from the adverse effects typically associated with lower maternal education.

In addition, this study was conducted in a psychiatric clinical setting, where adolescents often present with severe mental health symptoms, including anxiety, depression, and SI. As such, the findings may not be directly generalizable to adolescents in non-clinical settings, such as schools or communities, where the prevalence and severity of SI are likely lower. Adolescents in psychiatric settings may experience heightened family stressors, more pronounced interpersonal difficulties, and more severe psychological symptoms, which could amplify the observed associations between family environmental factors and SI. For instance, the protective effect of positive RQ or intact parental marital status might be less pronounced in the general population, where baseline mental health is typically better. Similarly, the mediating role of anxiety in the pathway from family factors to SI might differ in non-clinical populations, where anxiety levels may not reach clinical thresholds.

This study has several limitations that should be considered when interpreting the findings. First, the sample consisted solely of adolescents seeking psychiatric care, which may limit the generalizability of the results to other adolescent populations, particularly those not engaged in psychiatric services. These individuals often exhibit more severe mental health symptoms, which could exaggerate the observed associations between family environmental factors and SI. Second, the cross-sectional design of the study restricts the ability to establish temporal or causal relationships, making any such interpretations speculative. While network analysis reveals associations, it does not account for temporal precedence, leaving the possibility of reverse causation. For instance, while poor family relationships may contribute to SI, it is equally plausible that adolescents with SI perceive their family relationships more negatively. Additionally, the reliance on self-report measures, despite using validated tools like HADS and SITBI, may introduce bias due to social desirability or inaccurate self-assessment, potentially impacting the validity of the findings. Finally, omitted variable bias cannot be ruled out, as unmeasured factors such as peer relationships or genetic predispositions may influence both family environment and SI.

## Data Availability

The original contributions presented in the study are included in the article/supplementary material. Further inquiries can be directed to the corresponding authors.
